# Potential Antitumor Effect of Functional Yogurts Formulated with Prebiotics from Cereals and a Consortium of Probiotic Bacteria

**DOI:** 10.3390/foods12061250

**Published:** 2023-03-15

**Authors:** Alexandru Ciric, Nicoleta Radu, Marilena Gabriela Olteanu Zaharie, Georgeta Neagu, Lucia Camelia Pirvu, Mihaela Begea, Amalia Stefaniu

**Affiliations:** 1 Faculty of Biotechnical Systems Engineering, University Politehnica of Bucharest, 313 Splaiul Independentei, 060042 Bucharest, Romania; 2 ICA Research and Development S.R.L, 202 Splaiul Independentei, 060021 Bucharest, Romania; 3 Faculty of Biotechnology, University of Agronomic Sciences and Veterinary Medicine of Bucharest, 59 Marasti Boulevard, 011464 Bucharest, Romania; 4 Department of Biotechnology, National Institute of Chemistry and Petrochemistry R&D of Bucharest Romania, 202 Splaiul Independentei, 060021 Bucharest, Romania; 5 Department of Pharmaceutical Biotechnology, National Institute for Chemical Pharmaceutical R&D of Bucharest, 112 Calea Vitan, 031299 Bucharest, Romania

**Keywords:** yogurts, red rice, malts, postbiotics antitumor

## Abstract

Various types of functional yogurts were obtained from normalized milk (with normalized lipid content) and a standardized probiotic consortium of probiotic bacteria named ABY3. All the types of yogurts obtained contained prebiotics from black or red rice; malt of barley, rye, wheat; or wheat bran. The physico-chemical analyses of all the functionalized products obtained showed that all of them met the quality standard for yogurt products. However, the sensorial analyses showed that the products obtained from black and red rice were of very good quality. The biological analyses indicated that all the types of products contained live probiotic bacteria, but wheat bran and red rice could increase their numbers. Tests performed on tumor cell line Caco-2 with corresponding postbiotics revealed cytotoxicity greater than 30% after 48 h of exposure in the case of yogurts obtained from milk with 0.8% lipid content and red rice or blond malt of barley. In the case of yogurts derived from milk with 2.5% lipid content, only the variants that contained blond malt of rye or wheat became cytotoxic against the Caco-2 cell line.

## 1. Introduction

The human digestive system contains billions of organisms and is considered the ecosystem with the densest population of bacteria, yeasts, fungi, and viruses. All these species and their genomes form the gut microbiome [1]. This ecosystem is often compared to a bioreactor, which, when in balance, positively influences metabolic processes, tissue development processes, and immunomodulatory processes, especially in the presence of species of the *Bifidobacterium* or *Lactobacillus* genus [2,3]. The existence of some imbalance in the functioning of this bioreactor leads to the appearance and maintenance of the disease state [4,5], especially when chemotherapeutic treatments are used. The studies carried out until now suggest that the gut microbiome is responsible for the appearance and maintenance of diseases such as cancer [6,7]. According to the World Health Organization, in the year 2020, digestive cancers (colon, stomach, liver) represented the main causes of death. According to data published in the year 2020, 10% of the patients were diagnosed with colorectal cancer and 9.4% of existing patients with this condition died; 5.6% of new patients were diagnosed with stomach cancer and 7.7% of the existing patients with this condition died; and 4.7% of patients were diagnosed with liver cancer and 8.3% of patients with this condition died [8].

Further research has indicated the importance of *Bifidobacterium* in the immune response and the respective response to different types of antitumor therapies [8,9,10]. Thus, the aqueous extracts of inactivated probiotic microorganisms from the *Bifidus* and *Lactobacillus* genera exhibit cytotoxicity against the tumor cell lines of type MKN-1 (human gastric tumor cell line) [9]. The paraprobiotics or postbiotics used in the above-mentioned studies came from *B. bifidum*, *B. breve*, *L. bulgaricus*, *L. casei*, *L. rhamnosus*, *L. gasseri*, *L. plantarum*, and *L. reuteri* [9]. During a life cycle, commensal bacteria produce low-molecular-mass metabolites; these compounds play an essential role in regulating growth and reproduction processes, stimulating the growth processes of other microorganisms, influencing intercellular communication, and protecting against stress factors [11,12,13,14,15]. Many of these soluble metabolites can be secreted by living bacteria (bacterial metabolites) or released during bacterial lysis, providing physiological benefits to the host. These postbiotics differ either in their elemental composition (lipids, proteins, carbohydrates, vitamins, and organic acids) or in their biological activity (immunomodulatory, anti-inflammatory, hypocholesterolemic, antiobesogenic, antihypertensive, antiproliferative, or antioxidant activity) [16,17,18,19,20]. The presence of postbiotics in the digestive system inhibits the development of pathogenic bacteria such as *Listeria monocytogenes*, *Salmonella enterica* S-100, and *Escherichia coli* E-30, as well as some *Enterococcus* species resistant to antibiotics [16,18,20]. Moreover, an antioxidant effect was highlighted, both in vitro and in vivo, in certain metabolites exposed to specific exopolysaccharides of *Bifidobacterium animalis* strains, which succeeded in inhibiting oxide and superoxide radicals [21,22,23]. The gut microbiome allows complex nutrients, such as the cell wall components in plants [24,25,26,27] or cereals (cellulose, pectin, hemicellulose, lignin, mucins, and polysaccharides (prebiotics)), to be transformed into simple sugars that are fermented to form short-chain fatty acids (SCFAs). These compounds (SCFAs) contain organic compounds such as acetate, propionate, and butyrate and are usually amplified by the activity of the bioactive compounds that exist in fractions isolated from plants [28,29] or cereals, such as polyphenolic compounds [30,31,32], which are easily highlighted with Folin–Ciocalteu reagent [29]. Organic acids, mainly SCFAs, are formed in the gastrointestinal tract in millimolar amounts and occur in particular in large amounts in those areas where anaerobic microorganisms are predominant. The main probiotic microorganisms with a role in the production of SCFAs with cytotoxic properties [33,34] with the ability to inhibit the colorectal tumor cell lines are the following:

- *Bifidobacterium* sp., *Blautia hydrogentrophica*, *Prevotella* sp., and *Streptococcus* sp. produce through their own metabolism acetic acid, propionic acid, and lactic acid as SCFAs [35,36,37,38];
- *Lactobacillus salivarius* sbsp. *salcinius* JCM 1230, *Lactobacillus agilis* JCM 1048, *Lactobacillus rhamnosus* GG (LGG), *Lactobacillus gasseri* PA 16/8 and *Lactobacillus acidophilus* CRL 1014 produce through their own metabolism acetic acid, propionic acid, butyric acid, and lactic acid [39,40,41,42,43,44,45].

**Figure 1:**
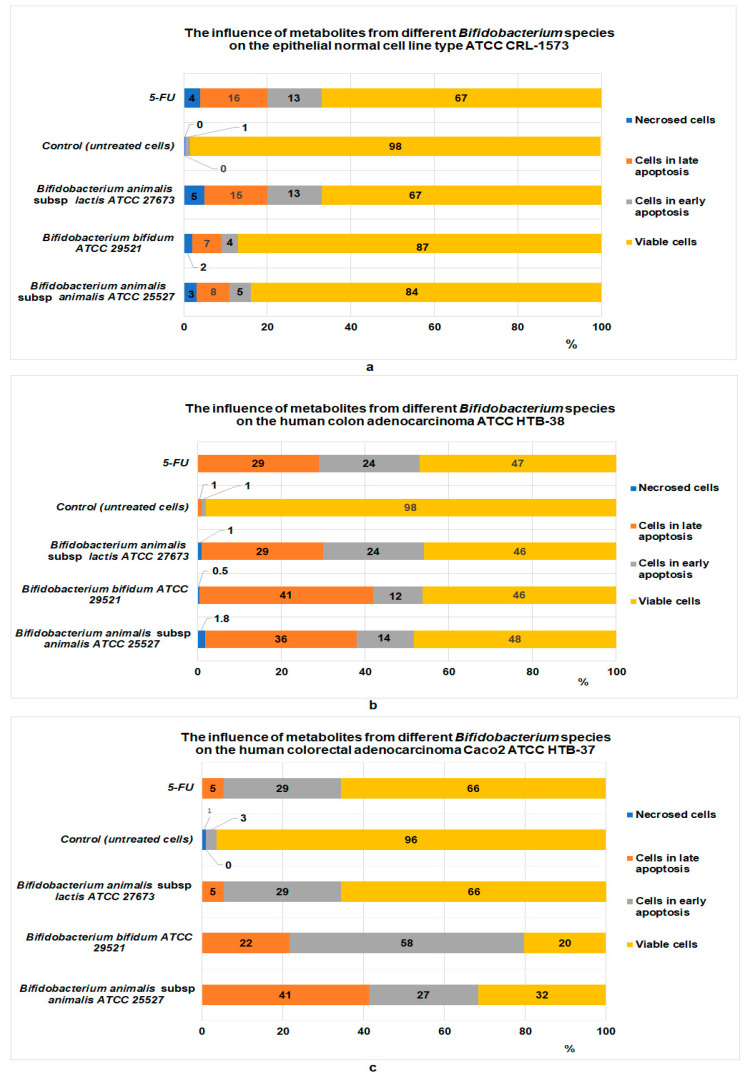
The effect of *Bifidobacterium* sp. metabolites on different tumor cell lines (adapted after Faghfoori et al. [38]): (**a**) effect on normal epithelial cell line CRL-1673; (**b**) effect on colon adenocarcinoma cell line of type HTB-38; (**c**) effect on colorectal adenocarcinoma Caco-2-HTB-37.

Li et al. [46] reported that in the case of patients with colorectal cancer, *Streptococcus thermophilus* is absent from their own gut microbiome, and this is a possible reason why tumor cells proliferate. The studies carried out by Uriot et al. [47] in an experimental model regarding the human digestive system showed that genetically modified *Streptococcus thermophilus* can survive under stress conditions inside of the human digestive system (i.e., stomach, duodenum, jejunum, and ileum). In addition, *Streptococcus thermophilus* can adhere to Caco-2-TC7 tumor cells by modulating their own surface properties. Sue Yue et al. [48], in studies carried out with different species of *Lactobacillus* (*L. acidophilus*, *L. plantarum*, and *L. rhamnosum*), showed that their metabolites can inhibit the development of tumor cells such as HT-29, IEC-6, or Caco-2. In an experiment carried out in vitro by Faghfoori et al., the tumor cells of types HTB-38 and Caco-2-HTB-37 were exposed to the metabolites obtained from different species of *Bifidobacterium* [38], and the results (Figure 1a–c) showed that the metabolites produced by the bacteria of the *Bifidobacterium* genus can inhibit the proliferation of HT-29-type tumor cells and Caco-2.

**Figure 2:**
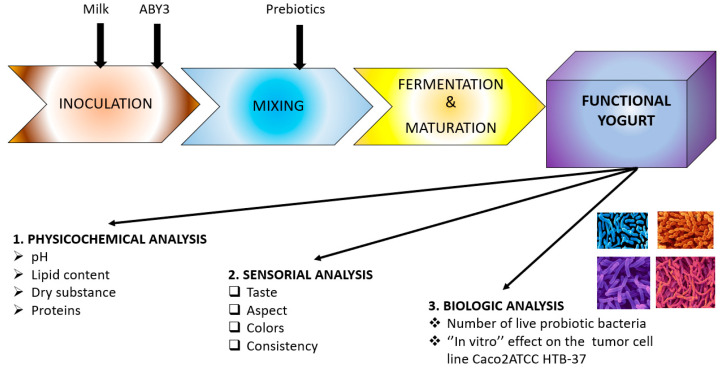
The methodology used in the experimental studies.

Taking all this into account, the research study we carried out aimed to obtain and characterize new types of yogurts containing both prebiotics and a consortium of standardized probiotic microorganisms. The characterization of these new products was made from three points of view (Figure 2): physicochemical (quality analyses specific to yogurt), organoleptic (sensorial analyses), and biological (the number of live probiotic bacteria in each product and the influence of each corresponding postbiotic on colorectal tumor cells of type Caco-2-ATCC-HTB-37).

## 2. Materials and Methods

### 2.1. Raw Materials Used for Obtaining Yogurts with Prebiotics

#### 2.1.1. Milk Source

Milk from an individual producer was used as the main raw material to obtain different types of yogurts. The quality characteristics of the milk used are presented in Table 1. The selected milk was normalized according to the methodology presented by Sadeghi Vasafi and collab. [49] from the point of view of lipid content. In this way, we have obtained two types of yogurts:

(1) Yogurts with low lipid content;
(2) Yogurts with normal lipid content.

#### 2.1.2. Prebiotic Sources

As sources of prebiotics, we used finely ground materials (granulation of <0.1 mm) obtained from black rice, red rice, barley malt, rye malt, wheat malt, and wheat bran, used in an MM400-type ball mill, RETSCH (Retsch GmbH, Haan, Germany). The characteristics of the prebiotic sources are presented in Table 1. The three types of malts were purchased from Weyermann Specialty Malting (Bamberg, Germany), and the content of dry substances and humidity was supplied by the seller (Table 1).

##### Moisture Content

The moisture contents in cereals and cereal products (rice, wheat, and bran) were determined according to the reference method by drying the samples at 130–133 °C for two hours. The moisture contents in malts (wheat, barley, and rye malts) were determined according to the EBC 4.2 method by drying the samples at 105–106 °C for three hours. As equipment, a Memmert UN55 drying oven (Schwabach, Germany) and an analytical balance (SHIMADZU ATX224R; Kyoto, Japan) were used.

##### Fiber Content

The determination of total dietary fiber contents in the samples of cereals, malts, and yogurts was performed using the gravimetric–enzymatic method (Official Methods AOAC 991.42 and AOAC 985.29) using a dietary fiber analyzer composed of a VELP GDE enzymatic digester and a VELP CSF6 filtration unit (VELP Scientifica, Usmate, Italy), a MULTISTIRRER magnetic stirrer, and a heating circulating bath (both from VELP Scientifica, Usmate, Italy). In addition, the equipment used included a Gallenkamp classic oven (Cambridge, UK), a Memmert UN55 drying oven (Schwabach, Germany), a pH meter (Hanna HI2002; Hanna Instruments, Smithfield, VA, USA), and an analytical balance (OHAUS AX224M; OHAUS Corporation, Parsippany-Troy Hills, NJ, USA). The specific enzymes (α-amylase E-PANAA-12G, amyloglucosidase E-AMGDF-100ML, and protease E-BSPRPD) were obtained from Megazyme International Ireland Limited (Bray, Ireland). Celite 545 (particle size of 0.02–0.1 mm) was obtained from Merck (Darmstadt, Germany).

##### Protein Content

The determination of the protein contents in the cereals and cereal products was performed according to the Kjeldahl method, in accordance with the ISO 8968-1:2014 standard (IDF 20-1:2014). The determination of the protein contents in malts (wheat, barley, and rye malts) was performed according to the EBC 4.3.1 method. Crude protein was obtained based on the nitrogen content determined using the Kjeldahl method multiplied by the factor of 6.25. As equipment, a digestion unit (Buchi K-426; Labexchange, Burladingen Deutschland), a titration unit (Titrino Plus 877; Metrohm Analytics, Bucharest, Romania), and a distillation unit (KjelFlex 360; VWR International, Vienna, Austria) were used. In addition, an analytical balance (OHAUS AX224M; OHAUS Corporation, Parsippany-Troy Hills, NJ, USA) was used.

##### Carbohydrate Content

Total carbohydrates were calculated based on the percentage of ash, total fat, moisture, and protein content using Equation (1).

Carbohydrates (%) = 100 − Ash − Total Fat − Moisture − Protein (1)

##### Polyphenol Content

The contents of polyphenols in red rice and black rice were determined using the Folin–Ciocalteu method using the methodology presented by Bolea et al. [29]. The content of polyphenols of each type of yogurt was determined from the supernatant resulting after centrifugation at 5000 rpm, using a HETTICH Universal 320 centrifuge, supplied by S.C. Precisa SRL Sibiu, Romania. Each supernatant was sterilized by filtration, using funnels with membranes of 0.45 μm and 0.2 μm (products named postbiotics).

#### 2.1.3. Probiotic Source

As a source of probiotics, we used a standardized consortium of probiotic bacteria named ABY3, containing *Lactobacillus acidophilus*, *Bifidobacterium animalis* subsp. *lactis*, *Streptococcus salivarius* subsp. *thermophilus*, and *Lactobacillus delbruecki* subsp. *bulgaricus* (commercial standardized products obtained from Christian Hansen, Hørsholm, Denmark).

#### 2.1.4. Cell Line Source

Proliferation studies were made on standardized cell line type Caco-2-ATCC-HTB-37, supplied by S.C. BioZyme SRL, Cluj Napoca, Romania.

### 2.2. Physicochemical Analyses of Milk and Yogurt

The quality analysis of milk and yogurt was carried out according to the SR 3665/1999 standard. The sensory analysis was carried out according to SR 6345/1995. Quality parameters such as dry matter, lipid content, protein content, and carbohydrate content were determined according to the Romanian standards (i.e., SR 3665:1999; SR 6345/1995; ISO 26323:2009; ISO 8968-1:2014; ISO 488:2008; ISO 13580; ISO/TS 22113:2012).

#### 2.2.1. Product Acidity

The acidity of the finished products (yogurts) was measured directly, using the portable pH meter Hanna H199161 (Hanna Instruments Inc., Smithfield, VA, USA).

#### 2.2.2. Solid Content

The determination of total solid contents in the samples of milk and yogurt was performed using the reference method of ISO 13580:2005|IDF 151:2005. As equipment, a Memmert UN55 drying oven (Schwabach, Germany), an analytical balance (SHIMADZU ATX224R; Kyoto, Japan), and a water bath (Memmert WB7-10; Schwabach, Germany) were used.

#### 2.2.3. Lipid Content

The determination of lipid contents in the samples of milk and yogurt was performed via the acido-butyrometric (Gerber) method using a Super Vario N centrifuge for butyrometers and specific butyrometers (Funke-Gerber, Berlin, Germany). Briefly, the fat content, expressed as a percentage, was read directly on the graduated scale of the butyrometer, after having previously dissolved the protein substances by treating the sample with sulfuric acid and separating the fat by means of centrifugation in the presence of a small amount of amyl alcohol. In the case of yogurt, the method of the undiluted sample was applied. An analytical balance (SHIMADZU ATX224R; Kyoto, Japan) and a water bath (Memmert WB7-10; Schwabach, Germany) were used as equipment.

#### 2.2.4. Sensorial Analysis

Sensorial analyses were made according to standard SR 6345/1995. Ten people were put in a clean room, and each person received the 14 samples identified by a number from 1 to 14. These persons analyzed each sample and gave a value from 1 to 5 for each parameter analyzed for each type of yogurt. The size of each sample was a hermetically closed recipient with 200 mL of yogurt.

### 2.3. Biological Determination

#### 2.3.1. Determination of the Total Number of Live Lactic Bacteria

The content of live lactic acid bacteria in each variety of yogurt obtained was determined by weighing, under sterile conditions, one gram of the product, followed by mixing this amount with 10 mL of sterile distilled water. This suspension was used to obtain five serial dilutions with sterile distilled water (dilutions made: 1:10; 1:100; 1:1000; 1:10,000; and 1:100,000). Each dilution was used for inoculation in Petri dishes with agarized MRS. The inoculated plates were incubated at 37 °C for 24 h in an Ibx INC 65 incubator (Labbox, Barcelona, Spain). After 24 h, the number of formed colonies (CFU) was read, and the final result was reported in the form of logCFU. Each determination was made three times; the results were presented as average values with corresponding standard deviations.

#### 2.3.2. Evaluation of the Effect Induced by Obtained Yogurts on the Proliferation of Tumor Cell Line Caco-2-ATCC-HTB-37

In the proliferation studies, the supernatant obtained after centrifuging yogurt at 6000 rpm with a Hettich EBA 200S centrifuge (Andreas Hettich GmbH&KG Tuttlingen, Germany) was used. Before use, the supernatant was sterilized by means of filtration with 0.45 μm membranes and then with membranes of 0.2 μm. The sterile supernatant, called postbiotic, was used to supplement the culture medium of Caco-2-type tumor cells. Proliferation studies were performed in microplates with 96 wells, using the MTT method [50,51,52] on the Caco-2-ATCC-HTB-37tumor cell line. Readings were performed at 24 and 48 h, using a Biobase BK-EL10C plate reader (Solantis, Bucharest Romania), at 492 nm [50,51,52]. The proliferation index (PI) was calculated according to relation 2.

(2) $$\mathrm{PI}\;=\frac{\mathrm{Vi}}{\mathrm{Vo}}\;$$

Here, Vo represents the % of the viability of the control (untreated cells, considered as the negative witness), and Vi represents the viability of the experimental variant. As a positive witness, we considered the postbiotics obtained from yogurt without prebiotic content.

### 2.4. Statistical Analysis

All determinations were made in triplicate, and the results are presented as an average of these. All determinations were made in three repetitions; the graphs were made representing the average of the three determinations, with corresponding standard deviations. The obtained data from studies performed in vitro on the cell line were analyzed statistically with the Graph Pad Prism 5 program, in which the values were compared to the negative control. The statistical significance of the obtained results was made as a function of the value of the parameter “*p*” generated by the statistical program, which is represented on the graph with notations: NS, *, **, or ***, with the following statistical significances:

Notation: NS = results without statistical significance, i.e., *p* > 0.05;
Notation: * = results with statistical significance, i.e., 0.05 < *p* < 0.01;
Notation: ** = results with distinct statistical significance, i.e., 0.01 ≤ *p* < 0.005;
Notation: *** = results with high statistical significance, i.e., *p* ≤ 0.005.

## 3. Results

### 3.1. Physicochemical Analysis

**Figure 3:**
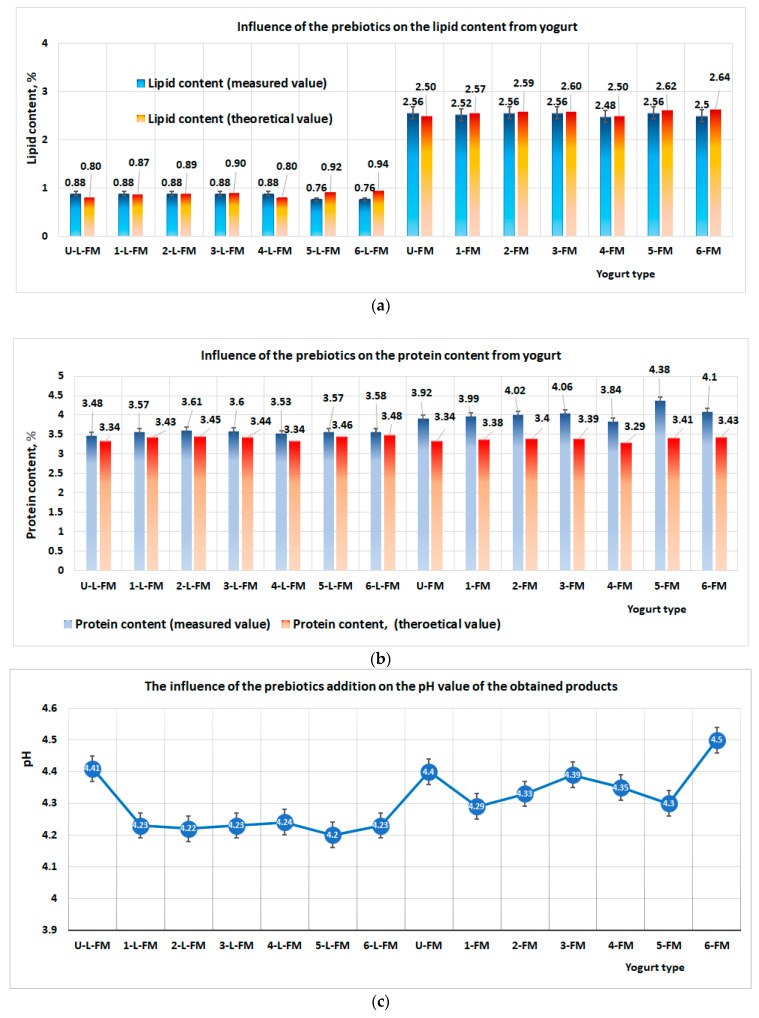
Results of physical–chemical analysis of products obtained with a consortium of prebiotic bacteria and prebiotics: (**a**) influence of prebiotics on lipid content in yogurt; (**b**) influence of prebiotics on protein content in yogurt; (**c**) influence of prebiotic addition on the final pH of products.

Regarding the content of lipids, the measured values of all the samples were different from those of the main raw material (milk). These differences were due to the prebiotic content, which comes with its own content of lipids (Table 2), so the end values were in fact the sum of the lipid content in milk and each prebiotic material (Figure 3a). In the case of the protein content in the yogurt products, the analyses performed showed that the measured values were different from the theoretical values obtained from the sum of raw materials (Figure 3b), i.e., milk and prebiotics. The contents of protein in the products obtained ranged between 4% and 4.38% in the case of yogurt obtained from milk with 2.5% lipids. In the products obtained from milk with 0.8% lipid content, the protein contents ranged between 3.48% and 3.61%. Regarding the acidity of the products, a slight variation was obtained in the two types of yogurts (Figure 3c). In the case of yogurt obtained from milk with 0.8% lipid content, the product pH decreased from 4.41 (yogurt without prebiotic content) to 4.2. The presence of prebiotics decreases the pH value of the end products, probably due to probiotic microorganisms that metabolize the glycosidic compounds from prebiotics. In the case of the products obtained from milk with 2.5% lipid content, the pH of the yogurts ranged between 4.5 and 4.29. The highest pH (4.5) was obtained in the products that contained wheat bran (Figure 3c). The data obtained from all types of yogurt analyzed (Table 2) showed the following:

- The pH parameter had values in the range of 4.22 and 4.5, meeting the standard SR 3665/1992
- The contents of protein in products obtained from semi-skimmed milk ranged between 3.48% and 4.38%, meeting the standard SR 3665/1992;
- The contents of lipids in bioproducts ranged between 0.76% and 2.56, meeting the standard SR 3665/1992;
- The content of dry substances ranged between 11.17% and 12.23%, meeting the standard SR 3665/1992 for this parameter.

### 3.2. Sensorial Analysis

**Figure 4:**
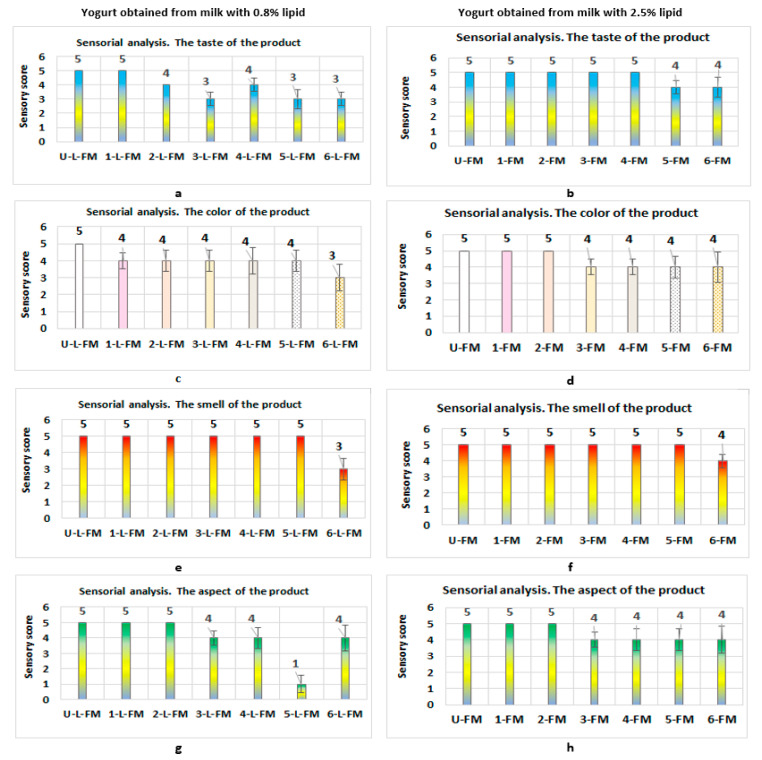
Sensorial analysis of yogurt obtained from milk and/or prebiotics: (**a**,**b**) the taste of the products; (**c**,**d**) the color of the products; (**e**,**f**) the smell of the products; (**g**,**h**) the aspect of the products. The untreated product and the product which contained black rice obtained the best scores in the sensorial analysis.

The sensorial analyses performed on the bioproducts obtained from milk with 0.8% lipid content and from milk with 2.5% lipid content (Figure 4a–h) showed the following:

(1) In the case of bioproducts obtained from milk with a content of 0.8% lipids, the best organoleptic properties were obtained in the case of product U-L-FM (yogurt without prebiotics), followed by products that contained black rice (1-L-FM), red rice (2-L-FM), and blond malt of rye (4-L-FM) (Figure 4a,c,e,g)

Low values of the organoleptic parameters of “taste”, “color”, and “smell” were found for product 6-L-FM (value received = 3). Regarding the aspect of the product, the lowest value (score = 4) was found for product 5-L-FM, obtained from blond malt of wheat. Regarding the “color” parameter, moderate values (score = 4) were obtained in the case of bioproducts with different prebiotic contents because customers obviously know that defatted yogurt is not colored. Despite the moderate value obtained for the color parameter, however, regarding the “aspect” organoleptic parameter, the products with black rice (1-L-Fm) and red rice (2-L-FM) received the maximum value (score = 5).

(2) Regarding the yogurt products obtained from milk with 2.5% lipid content, the sensorial analysis showed the following:
  - Bioproducts U-FM, 1-FM, and 2-FM received the maximum score (5 points) for the organoleptic parameters of “taste”, “color”, “smell”, and “aspect” (Figure 4b,d,f,h respectively). Products 3-FM and 4-FM received the maximum score (5 points) for the parameter of “taste”, whereas products made with wheat malt and wheat bran (5-FM and 6-FM, respectively) received only 4 points.
  - All the products with malt as well as the product with wheat bran received a score of 4 points for the organoleptic parameters of “colors” and “aspect”.
  - In the case of the parameter of “smell”, the maximum score (5 points) was by products obtained with malt (3-FM; 4-FM; 5-FM).

### 3.3. Biological Analyses

#### 3.3.1. Influence of Prebiotics on the Content of Live Probiotic Bacteria

**Figure 5:**
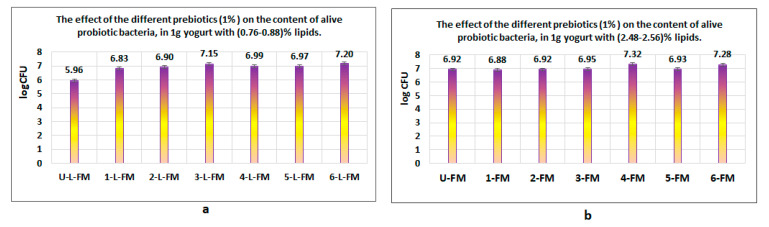
Influence of prebiotic additions on the number of live probiotic bacteria in obtained products after maturation: (**a**) influence of prebiotic additions on the number of live probiotic bacteria in products containing (0.76–0.88)% lipids; (**b**) influence of prebiotic additions on the number of live probiotic bacteria in products containing (2.48–2.56)% lipids. A favorable effect on the multiplication of probiotic bacteria was obtained in the presence of wheat bran fibers in both yogurt types (i.e., yogurt with low lipid content and yogurt with normal lipid content).

Given the analysis of the data obtained, we can conclude that, in general, the presence of wheat bran in both types of products had a favorable effect on live probiotic bacteria (Figure 5a,b). However, some differences appeared as a result of the lipid content of raw material. In the case of yogurt with a low content of lipids (i.e., yogurt with (0.76 ÷ 0.88)% lipid content), the analysis revealed that the best influence on the number of live probiotic bacteria was obtained in the presence of wheat bran (logCFU = 7.2), followed by blond malt of barley (logCFU = 7.15) (Figure 5a).

Regarding the rest of the yogurt products, the number of probiotics decreased in the following order: yogurt without prebiotics < yogurt with black rice < yogurt with red rice < yogurt with blond malt of wheat < yogurt with blond malt of rye.

#### 3.3.2. Product Effect on Tumor Cell Line Caco-2

**Figure 6:**
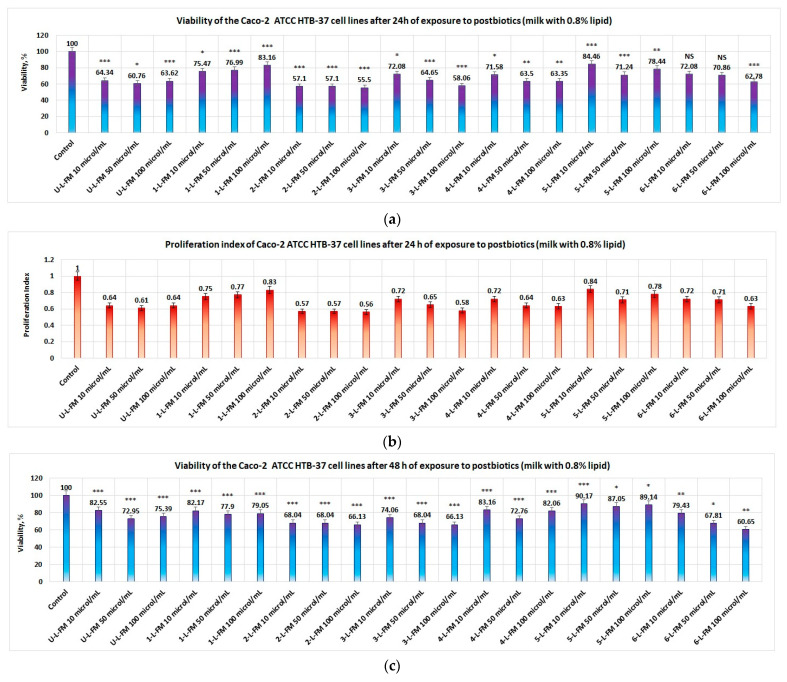

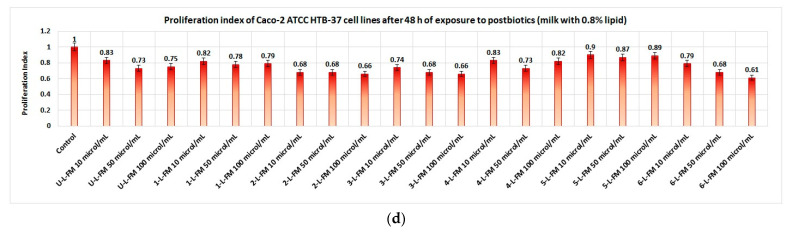
Influence of postbiotics obtained from yogurt with low content of lipids on Caco-2 proliferation: (**a**) cell line viability after 24 h of exposure to postbiotics; (**b**) proliferation index of Caco-2 cell line after 24 h of exposure to probiotics; (**c**) cell line viability after 48 h of exposure to postbiotics; (**d**) proliferation index of Caco-2 cell line after 48 h of exposure to probiotics. Postbiotics from yogurt obtained with prebiotics from red rice decreased the viability of the Caco-2 cell line after 24 and 48 h of exposure at all studied concentrations. Postbiotics from products with blond malt of barley at a concentration greater than 50 μL/mL decreased the proliferation of the Caco-2 cell line. Postbiotics from yogurt with wheat bran, after 48 h of exposure, decreased the proliferation of the Caco-2 cell line at a concentration greater than 50 μL/mL. Notation: NS = results without statistical significance, i.e., *p* > 0.05; Notation: * = results with statistical significance, i.e., 0.05 < *p* < 0.01; Notation: ** = results with distinct statistical significance, i.e., 0.01 ≤ *p* < 0.005; Notation: *** = results with high statistical significance, i.e., *p* ≤ 0.005.

The studies performed on postbiotics obtained from milk with 0.8% lipid content indicated that the majority of the data obtained are statistically significant (except samples 6-L-FM10 and 6-L-FM50) and also revealed the following:

- At 24 h, the lowest viability of the Caco-2 cell line was obtained in the case of postbiotics from yogurt formulated with red rice (2-L-FM). In this case, cell viability at concentrations of postbiotics in the range of (10–100) μL/mL was less than 57.1% (Figure 6a); the corresponding proliferation index (PI) was less than 0.57 (Figure 6b);
- In second place were the postbiotics obtained from milk with 0.8% lipid content without prebiotics; the viability of tumoral cells was less than 64.4%, and the corresponding proliferation index was less than 0.64 (Figure 6a,b). If the concentration of postbiotics was greater than 50 μL/mL, then the viability of the tumor cells was less than 65% in the case of products 3-L-FM and 4-L-FM, and the corresponding proliferation index was less than 0.65 (Figure 6a,b, respectively).

Low viability was also obtained in the case of product 6-L-FM at a concentration of postbiotics greater than 100 μL/mL (viability = 62.78% and proliferation index of 0.63). After 48 h of exposure to postbiotics, low values of viability were maintained only in the case of product 2-L-FM (viability of less than 68.1% and proliferation index of less than 0.681 (Figure 6c,d, respectively). A low level of viability was observed for product 3-L-FM at concentrations greater than 50 μL/mL (viability of less than 68.1% and proliferation index of less than 0.681) as well as for the postbiotics of 6-L-FM (viability of less than 67.9% and proliferation index of less than 0.68), in the same range of postbiotic concentration (Figure 6c,d).

**Figure 7:**
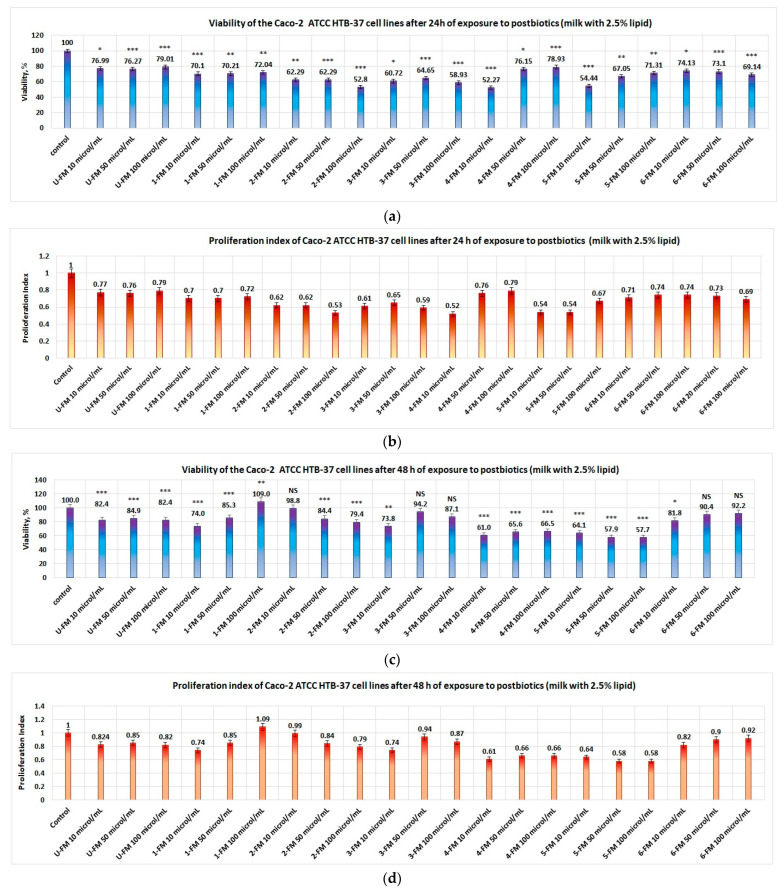
Influence of postbiotics obtained from yogurt with normal content of lipids on Caco-2 proliferation: (**a**) cell line viability after 24 h of exposure to postbiotics; (**b**) proliferation index of Caco-2 cell line after 24 h of exposure to probiotics; (**c**) cell line viability after 48 h of exposure to postbiotics; (**d**) proliferation index of Caco-2 cell line after 48 h of exposure to probiotics. Postbiotics from yogurt obtained with prebiotics from red rice and blond malt barely decreased the viability of the Caco-2 cell line after 24 h of exposure at all studied concentrations. Postbiotics from products with blond malt of rye and blond malt of wheat at all concentrations decreased the proliferation of the Caco-2 cell line after 48 h of exposure. Notation: NS = results without statistical significance, i.e., *p* > 0.05; Notation: * = results with statistical significance, i.e., 0.05 < *p* < 0.01; Notation: ** = results with distinct statistical significance, i.e., 0.01 ≤ *p* < 0.005; Notation: *** = results with high statistical significance, i.e., *p* ≤ 0.005.

In the case of postbiotics derived from yogurt obtained from milk with 2.5% lipid content, the data obtained are generally statistically significant, except the data obtained at 48 h for samples 2-DFM10; 3Fm50; 3-FM100; 6-FM50; and 6-FM100, and showed the following:

- If the tumor cells were exposed for 24 h to postbiotics, then the viability obtained in the case of cells treated with 2-FM was less than 62.3% at all concentrations studied, and the proliferation index obtained in this case was less than 0.63% (Figure 7a,b, respectively);
- Appropriate results were obtained in the case of product 3-FM: the viability of tumor cells was less than 64.7%, and the proliferation index was less than 0.66 (Figure 7a,b, respectively). In the case of the postbiotics resulting from product 5-FM, at concentrations greater than 50 μL/mL in the culture medium, the viability of tumor cells was under 55%. Additionally, upon exposure to postbiotics derived from products 4-FM and 6-FM (at a concentration greater than 100 μL postbiotics/mL), the viability of tumor cells was less than 52.3% and 69.2%, respectively (Figure 7a,b, respectively). By increasing the exposure time to 48 h, lower values of tumor viability were only obtained in the case of the postbiotics derived (Figure 7c,d).

**Figure 8:**
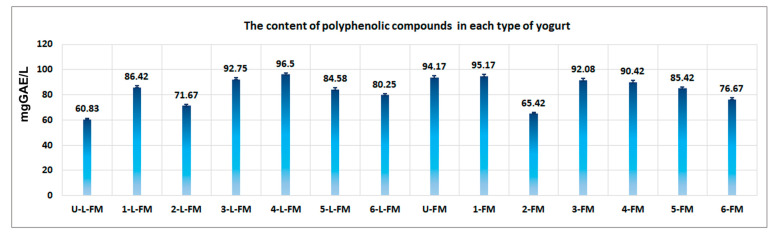
The content of polyphenolic compounds in each type of yogurt. A great level of polyphenolic compounds were found in yogurt 4-L-FM (96.5 mgGAE/L) obtained from blond malt of rye and milk with 0.8% lipid and in yogurt 1-FM (95.17 mgGAE/L) obtained from milk with 2.5% lipid.

Regarding polyphenolic compounds, the values obtained for the analyzed samples ranged between 60.83 mgGAE/L and 95.17 mgGAE/L (Figure 8). A relatively high level of polyphenolic compounds was found in sample 4-L-FM, which contained 96.5 mgGAE/L (yogurt formulated with blond malt of rye and milk with 0.8% lipid), and also in the postbiotic derived from the yogurt named 1-FM, which contained 95.17 mgGAE/L (yogurt formulated with black rice, from milk with 2.5% lipid). A high value was found in the postbiotic from the yogurt named U-FM which contained 94.17 mg GAE/mL (product formulated with milk with 2.5% lipid, without cereals), in product 3-L-FM which contained 92.75 mgGAE/L (formulated with milk with 0.8% lipid and blond malt of barley), and finally in the postbiotic derived from 4-FM which contained 90.42 mgGAE/L (formulated with milk with 2.5% lipid and blond malt of rye).

## 4. Discussion

**Figure 9:**
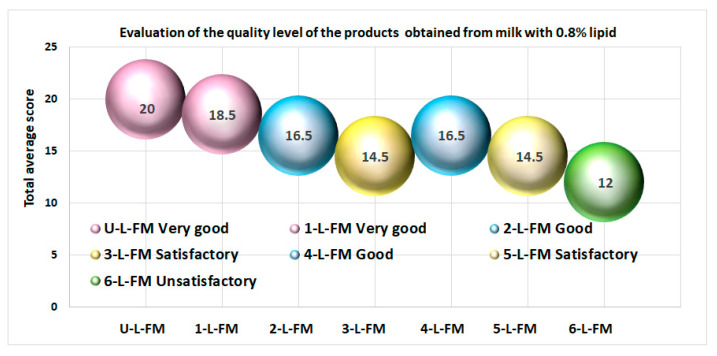
The quality level of yogurts with prebiotics obtained from milk with 0.8% lipid content was based on the results of the sensorial analyses. Only two products obtained the maximum quality level.

**Figure 10:**
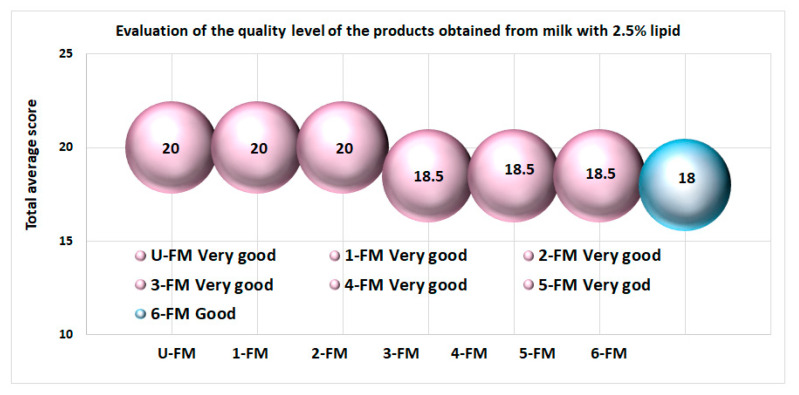
The quality level of yogurts with prebiotics obtained from milk with 2.5% lipid content was based on the results of the sensorial analyses. Most products obtained the maximum quality level, except for the product that contained bran of wheat.

First of all, in the case of products marketed for human use, commitment to the quality standards of the products is obligatory, and all the products obtained respected these standards. Regarding customer choices, sensorial analysis plays a major role. From this point of view, if we represent the quality level as a function of the sensorial analysis results (Figure 9 and Figure 10) (according to the SR 6345/1995 standard), then the results obtained show the following:

- In the case of yogurts with prebiotics obtained from milk with 0.8% lipid content, the qualificative “very good” was only obtained in the case of products U-L-FM (yogurt without prebiotics) and 1-L-F-M (yogurt that contained prebiotics from black rice) (Figure 9); most probably, these would be among the top customer preferences. In second place are the products named 2-L-FM (yogurt with red rice) and 4-L-FM (yogurt with blond malt of rye). The rest of the products with prebiotics obtained the qualificative “Satisfactory” or “Unsatisfactory”.

The main products obtained from milk with 2.5% lipid content obtained an average score greater than 18.1 (Figure 10), and all received the qualificative “Very good”. In the case of product 6-FM (yogurt containing prebiotics from the bran of wheat), the score obtained was less than 18.1, which corresponds to a quality level of “Good”. Most probably, if these products were marketed, they would be preferred by customers due to their organoleptic properties.

The level of polyphenolic compounds found in products with or without cereals is similar to the results obtained by Arafoui et al. [53] which reported a level of 8.5 mg GAE/100 g product in the yogurt formulated as a control. Other authors formulated yogurts with a high content of polyphenolic compounds by adding some subproducts which resulted after squeezing citrus fruits, strawberries, mulberry, dragon fruit, or passiflora [54,55,56,57,58,59]. In this case, the concentration of polyphenolic compounds ranged between 0.56 mgGAE/g and 1.28 mgGAE/g for yogurts with citrus [54] and between 20 mgGAE/mL and 64.43 mgGAE/mL for yogurt formulated with dragon fruit [55]. In this last case, it is important to mention that the yogurt formulated with dragon fruit used probiotic bacteria supplied by Cristian Hansen [59]. Regarding the content of live bacteria from yogurt products, the results obtained using this medium could have been influenced by the fact that *Streptococcus thermophilus* grows better on a specific medium, namely, M17 broth [60,61]. However, taking into account that all determinations were made in the same way, all the results contain the same error and do not appear to be influenced by this when compared. Various bibliographic sources indicate that in the MRS culture medium, species from the *Lactobacillus* and *Bifidobacterium* genera grow well [62,63,64]. Regarding yogurt with a normal content of lipids (i.e., yogurt with 2.48-2.56 % lipid content), the best influence on the number of live probiotic bacteria was obtained in the presence of blond malt of rye (lgUFC = 7.32) and wheat bran (lgUFC = 7.28) (Figure 5b).

If we assume that the values of the cytotoxicity of postbiotics after 48 h of exposure can be approximated by a first-degree polynomial, then the value of IC50 (IC50 = the concentration of selected postbiotics at which 50% of the live population of individuals is destroyed) obtained in all cases (Table 3) reveals that these products showed a moderate cytotoxicity level against the Caco-2 cell line, according to the NCI guidelines [33,65].

According to the studies performed by Gerhäuser [65], the cytotoxicity against the Caco-2 cell line of postbiotics derived from yogurts that contained prebiotics from blond malt of rye or blond malt of wheat was probably due to polyphenolic compounds in these materials, such as benzoic acid derivatives, cinnamic acids, coumarins, flavan-3-ols (catechins), proanthocyanidins, and chalcones, i.e., compounds with recognized activity against tumor cells. The antitumor mechanism of these compounds, which are also found in beer (which is obtained from different types of malt), implies angiogenesis suppression and inhibition of DNA synthesis in tumor cells. According to other studies [66,67], the enzymes contained in blond malt may also exhibit antitumor properties (the main enzymes from malts are alpha- and beta-amylases) [66,67]. The antitumor mechanism of these enzymes is based on the inhibition of glucose transporters (i.e., the suppression of glucose uptake in tumor cells). In addition, Jin et al. [68] found elevated contents of free phenolic compounds in barley (at concentrations ranging between 165 and 254 mg/100 g). The free phenolic compounds we found here were benzoic acids, cinnamic acid, hesperidin derivatives, and anthocyanins, which are compounds that are also found in malt produced from barley. The tumor activity of probiotics derived from yogurts that contained prebiotics from red rice could be due to the level of proanthocyanidins in red rice [69], which acts by altering the expression of the invasion-associated protein NF-kb [69].

Pereira-Caro et al. [70] found the following compound levels in red rice: anthocyanins, 1%; flavones and flavonols, 7%; gamma-oryzanol, 27%; and flavan-3-ols, 65%. Further, many of these compounds are being recognized for their antitumor activity [71]. Regarding the antitumor effect induced by the presence of probiotic microorganisms, the mechanism is all the more complex the greater the number of probiotic bacterial species. From this viewpoint, studies carried out in vitro in three colorectal cancer cell lines (HCT 116, HT 29, and Caco-2) compared with a normal colon epithelial cell line (MCM 4640) showed that *S. thermophillus* produces molecules of beta-galactosidase, which have the property of suppressing the development of tumor cells [46]. The antitumor effects of the *Lactobacillus* species are dominated by apoptosis and are induced by the accumulation of reactive oxygen species in the mitochondria, which leads to their destruction by means of the permeabilization of the external mitochondrial membrane. Membrane permeabilization leads to the irreversible release of proteins from the intermembrane space. This process leads to the sequential activation of Caspase-3, -8, and -9. The sequential activation of these types of proteins triggers the execution process of programmed cell death (apoptosis) [72,73]. Regarding probiotics from the *Bifidum* genus, the study we carried out showed that under the influence of *Bifidobacterium bifidum* metabolites, the inhibition process of tumor cells of type Caco-2-HTB-37 is dominated by early apoptosis (tumor cells in early apoptosis: 58%) (Figure 1c) and by late apoptosis (tumor cells in late apoptosis: 41%) in the case of HTB-38-type tumor cells (Figure 1b). Moreover, metabolites produced by *Bifidobacterium* sp. can down-regulate several genes involved in apoptosis suppression [46,62].

The results obtained are in agreement with research by Faghfoori and col. [38] in the case of *Bifidobacterium bifidum* metabolites’ effect on different colorectal tumor cell lines. According to their studies carried out using flow cytometry, tumor cell inhibition is achieved through a mechanism based on apoptosis. According to existing reported data, in the presence of postbiotics of *Bifidobacterium* sp., the viability of the studied tumor cells dropped below 50% (46% in the case of HTB-38 and 20% in the case of Caco-2-HTB-38) compared with the viability of normal epithelial cell lines, which was 87% (Figure 1a–c) [38]. The data reported by Faghfoori and col. are important for the present studies for two reasons:

(1) They explain the antitumor mechanism of postbiotics supplied by the microorganisms of the same genus (i.e., *Bifidobacterium*);
(2) In the consortium of probiotic microorganisms (standardized probiotic consortia, which contain thermophilic bacteria) exists *Bifidobacterium animalis* subsp. *lactis*, usually known as BB12; this microorganism appears to be identical to another microorganism supplied by the American Type Culture Collection, *Bifidobacterium animalis* subsp. *lactis* ATC 2763, and used by Faghfoori et al. in their studies.

Under the influence of postbiotics derived from *B. animalis* subsp. *lactis*, 29% of HTB- 37 tumor cells were found in early apoptosis and 5% were found in late apoptosis. In the case of the HTB-38 tumor cell line, exposed to the same postbiotics, 29% of the cells were found in the stage of late apoptosis and 24% in early apoptosis. In comparison, after the exposure of the normal cell line (ATCC CRL1575) to the same postbiotics, 15% of them were found in late apoptosis and 13% in early apoptosis.

## 5. Conclusions

Twelve types of yogurt products were obtained using milk with 2.5% lipid content or 0.8% lipid content as raw material, a commercial standardized consortium of probiotic bacteria (ABY-3), and different prebiotics in contents of less than 1% (black or red rice, barley, rye or wheat blond malt, and wheat bran). The quality analysis revealed that all obtained products met the quality standards of the legislation in force. The sensorial analysis indicated that in the case of yogurt products with a low content of lipids (less than 1%), only two products obtained the maximum quality level, whereas among the yogurt products with a normal content of lipids (greater than 2.5%), the main products obtained the maximum score.

Regarding biological activity, the study performed in vitro in the Caco-2 cell line with postbiotics derived from yogurt products revealed the cytotoxic activity of products 2-L-FM (product with prebiotics from red rice) and 3-L-FM (product with prebiotics from blond malt of barley) after 24 and 48 h of exposure, respectively. In the case of products with a normal content of lipids, the lipid content probably reduced the cytotoxic activity of the postbiotics; however, after 48 h of exposure, only the postbiotics derived from products 4-FM (product with prebiotics from blond malt of rye) and 5-FM (product with prebiotics from blond malt of wheat) became cytotoxic against the Caco-2 cell line. The mathematical modeling of the obtained results showed that the postbiotics with cytotoxicity greater than 30% showed moderate cytotoxic activity against the Caco-2 cell line; this fact suggests a protective activity of these types of foods against the Caco-2 tumor cell line. In the near future, other studies will need to be performed in order to evaluate the mechanism of action of selected postbiotics. Research should be initiated on standardized tumor cell lines derived from the human digestive system and should be aimed at establishing the following: the antioxidant activity of postbiotics; the influence of postbiotics on the cell cycle; and the levels of contribution of processes such as late apoptosis, early apoptosis, and necrosis in these tumor cell lines.

## Figures and Tables

**Table 1:** Physicochemical characteristics of the raw materials used for obtaining different types of functional yogurt.

MILK		pH	Lipid, %	Proteins, %	Dry Substance, %
Normalized milk with 0.8% lipids		6.67 ± 0.02	0.8 ± 0.01	3.34 ± 0.02	8.23 ± 0.03
Normalized milk with 2.5% lipids		6.61 ± 0.01	2.5 ± 0.2	3.29 ± 0.01	10.29 ± 0.01
**PREBIOTIC SOURCES**					
**RICE**	**CFU/g**	**Carbohydrates, %**	**Proteins, %**	**Polyphenols [29] mg GAE/g**	
1. Black rice	0	68.8 ± 0.1	7 ± 0.50	5.31 ± 0.10	
2. Red rice	0	79 ± 0.02	9.6 ± 0.10	3.7 ± 0.05	
**MALT OR BRAN**	**Humidity, %**	**Dry substance, %**	**Proteins, %**	**Fiber, %**	
3. Blond malt of barley	4.5 ± 0.05	82.1 ± 0.10	10.5 ± 0.05	6.89 ± 0.10	
4. Blond malt of rye	5.4 ± 0.06	82.7 ± 0.10	-	7.12 ± 0.12	
5. Blond malt of wheat	4.5 ± 0.05	82.9 ± 0.10	12.2 ± 0.10	6.94 ± 0.10	
6. Wheat bran	10.56 ± 0.1	-	14.38 ± 0.10	40.72 ± 0.12	

**Table 2:** Physicochemical analysis of yogurt products.

Yogurt Type			Code	Dry Substance, %
Untreated yogurt obtained from milk with 0.8% lipid			U-L-FM	10.14 ± 0.10
Yogurt obtained from milk with 0.8% lipid and 1% black rice			1-L-FM	10.85 ± 0.10
Yogurt obtained from milk with 0.8% lipid and 1% red rice			2-L-FM	11.29 ± 0.09
Yogurt obtained from milk with 0.8% lipid and 1% blond malt of barley			3-L-FM	10.94 ± 0.20
Yogurt obtained from milk with 0.8% lipid and 1% blond malt of rye			4-L-FM	10.52 ± 0.20
Yogurt obtained from milk with 0.8% lipid and 1% blond malt of wheat			5-L-FM	10.81 ± 0.20
Yogurt obtained from milk with 0.8% lipid and 1% wheat bran			6-L-FM	11.29 ± 0.20
Untreated yogurt obtained from milk with 2.5% lipid			U-FM	11.17 ± 0.05
Yogurt obtained from milk with 2.5% lipid and 1% black rice			1-FM	11.22. ± 0.17
Yogurt obtained from milk with 2.5% lipid and 1% red rice			2-FM	11.27 ± 0.20
Yogurt obtained from milk with 2.5% lipid and 1% blond malt of barley			3-FM	12.02 ± 0.18
Yogurt obtained from milk with 2.5% lipid and 1% blond malt of rye			4-FM	12.23 ± 0.07
Yogurt obtained from milk with 2.5% lipid and 1% blond malt of wheat			5-FM	12.32 ± 0.08
Yogurt obtained from milk with 2.5% lipid and 1% wheat bran			6-FM	11.22 ± 0.13
**Quality parameters according to Romanian legislation in force for partially skimmed yogurt** **(i.e., SR 3665:1999; SR 6345/1995; ISO 26323:2009; ISO 8968-1:2014; ISO 488:2008; ISO 13580; ISO/TS 22113:2012)**				
**pH**	**Proteins, %**	**Lipid, %**	**Dry substance, %**	
3.8–5.5	2.8%	(0.5–3)%	(8.5–11)%	

**Table 3:** Toxicity evaluation after 48 h of exposure of each product showing cytotoxic effects on the tumor cell line of type Caco-2 ATCC HTB-37.

Sample	IC50	Toxicity
2-L-FM	118.76	Moderate toxicity
3-L-FM	120.34	Moderate toxicity
6-L-FM	109.55	Moderate toxicity
2-FM	90.22	Moderate toxicity
3-FM	87.14	Moderate toxicity
4-FM	166.89	Moderate toxicity
5-FM	126.71	Moderate toxicity

Cytotoxicity scale: IC50 < 50 μL/mL high cytotoxicity activity; IC50 = (21–500) μL/mL: moderate cytotoxicity activity; IC50 > 500 μL/mL: no cytotoxicity.

## Data Availability

The data presented in this study are available upon request from the corresponding author.

## Abbreviations

Paraprobiotics or postbiotics	synonymous terms that define bioproducts obtained from non-viable microbial cells, raw microbial extracts, or inactivated probiotics.
Caco-2-TC7	standardized tumor cell line for human colon adenocarcinoma; this cell line is often used for studies performed in vitro regarding the permeability and absorption of bioactive compounds at the intestinal level.
Caco-2-ATCC-HTB-37	standardized human epithelial cells isolated from the colon tissue of a man with colorectal adenocarcinoma. This cell line has applications in cancer and toxicology research.
Epithelial normal cell line ATCC CRL-1573	standardized cell line with epithelial morphology isolated from the kidney of a human embryo, often used in industrial biotechnology and toxicology research.
Colon adenocarcinoma ATCC HTB-38	standardized human epithelial cell line isolated from a primary tumor obtained from a female patient with colorectal adenocarcinoma. This cell line has applications in cancer and toxicology research. Colorectal adenocarcinoma ATCC HTB-39: standardized human epithelial cells line isolated from colorectal adenocarcinoma of the large intestine (colon) with applications in cancer and toxicology research. Invasion-associated protein NF-kb is a protein complex that controls the transcription of DNA, cytokine production, and cell survival. These proteins are involved in cellular responses to stimuli such as cytokines, free radicals, heavy metals, and bacterial or viral antigens.
